# The association between the cutaneous sensory block area, the surgical incision’s location, and the block’s analgesic efficacy: a post hoc sensitivity analysis of data from a controlled randomised multicentre trial

**DOI:** 10.1007/s00464-025-11777-3

**Published:** 2025-05-09

**Authors:** Christopher Blom Salmonsen, Kai Henrik Wiborg Lange, Jakob Kleif, Rasmus Krøijer, Lea Bruun, Martynas Mikalonis, Peter Dalsgaard, Karen Busk Hesseldal, Jon Emil Philip Olsson, Claus Anders Bertelsen, the OPMICS Study Group, Jeanette Lydeking, Christina Kraiberg Rokatis, Uffe Schou Løve, Susie Lindhardt Larsen, Claudia Jaensch, Anders Husted Madsen

**Affiliations:** 1 Department of Surgery, Copenhagen University Hospital – North Zealand, Hillerød, Denmark; 2 Graduate School, Faculty of Health and Medical Sciences, University of Copenhagen, Copenhagen, Denmark; 3 Department of Anaesthesiology, Copenhagen University Hospital – North Zealand, Hillerød, Denmark; 4 Department of Clinical Medicine, Faculty of Health and Medical Sciences, University of Copenhagen, Copenhagen, Denmark; 5 Department of Surgery, Hospital of Southwest Jutland, Esbjerg, Denmark; 6 Department of Regional Health Research, Faculty of Health Sciences, University of Southern Denmark, Odense, Denmark; 7 Department of Surgery, Regional Hospital of Viborg, Viborg, Denmark; 8 Department of Surgery, Gødstrup Hospital, Herning, Denmark; 9 Department of Anaesthesiology and Intensive Care, Gødstrup Hospital, Herning, Denmark; 10 Department of Clinical Medicine, Faculty of Health, Aarhus University, Aarhus, Denmark; 11 Surgical Research Unit, Gødstrup Hospital, Herning, Denmark

**Keywords:** Pain management, Laparoscopy, Nerve block, Pain, Postoperative, Anaesthesia, Local

## Abstract

**Background:**

Transversus abdominis plane blocks are widely used, but the association between the cutaneous sensory block area and the analgesic effect is still debated. We aimed to determine the relationship between the cutaneous sensory block area, the surgical incision’s location, and the block’s analgesic efficacy.

**Methods:**

A sensitivity analysis of data from a multicentre, patient-, clinician-, investigator-blinded, placebo- and active-controlled, 3-arm randomised clinical trial. Patients undergoing minimally invasive colon surgery were included from four hospitals across Denmark between January 2021 and February 2024. In this sensitivity analysis, we used our previously collected data of the cutaneous sensory block area to examine the efficacy of two different approaches to the transversus abdominis plane block, as compared to each other and placebo based on incision location. The primary outcome was total morphine dose equivalents administered in the first 24 h after minimally invasive surgery in patients receiving either a Pfannenstiel or a supraumbilical transverse incision.

**Results:**

We found that the subcostal dual laparoscopic-assisted transversus abdominis plane block was superior to both the ultrasound-guided posterior transversus abdominis plane block and placebo in patients with a Pfannenstiel incision with an absolute difference of − 8.9 mg (95% CI, − 16.5 to − 1.3 mg; *p* = 0.02) and − 10.3 mg (95% CI, − 17.0 to − 3.6 mg; *p* < 0.01) morphine dose equivalents, respectively. No difference was found for the supraumbilical transverse incision. Patient-reported outcome measures favoured the laparoscopic-assisted block, with an absolute difference of 13 (95% CI, 1.7–24.3; *p* = 0.025) in the Quality of Recovery-15 score compared to placebo.

**Conclusion:**

The effect of the transversus abdominis plane block seems to be independent of the distribution of the cutaneous sensory block area of the approach. The laparoscopic-assisted subcostal transversus abdominis plane block reduced postoperative pain with a high QoR-15 score.

Transversus abdominis plane (TAP) blocks are currently recommended for postoperative pain management in minimally invasive colorectal surgery [1]. For TAP blocks, a bolus of local anaesthetic is injected into the plane between the transversus abdominis and internal oblique muscles. This aims to separate the fascial layers and allow for widespread diffusion, blocking as many cutaneous nerves as possible [2]. Studies using MRI or dye injection have suggested that TAP blocks, as field blocks, provide dermatomal coverage [3, 4]. In clinical settings, the cutaneous sensory block area (CSBA), measured as loss of sensation to cold, is often used to assess the presumed coverage of regional, field, and epidural blocks. We mapped the CSBA of three different TAP modalities: the ultrasound-guided posterior (US-TAP) block in healthy individuals [5], and the subcostal dual-TAP block applied ultrasound-guided [6] and laparoscopically guided (L-TAP) [7] in patients undergoing laparoscopic cholecystectomies. Our results question the assumption of TAP blocks’ dermatomal coverage and therefore their analgesic effect.

To definitively establish the analgesic effect of TAP blocks, we conducted optimal peripheral nerve block after minimally invasive colon surgery (OPMICS) [8], a multicentre, blinded, randomised controlled trial including 360 patients undergoing minimally invasive colon resections. OPMICS aimed to determine whether posterior US-TAP and subcostal dual L-TAP blocks reduce 24-h postoperative morphine equivalent consumption compared to placebo. The subcostal dual L-TAP block demonstrated statistical superiority to placebo and non-inferiority to the US-TAP block. However, neither block achieved the predetermined minimal clinically important difference of 10 mg morphine [9].

Figure 1 shows the combined CSBA maps of posterior US-TAP blocks in healthy individuals [5] and the subcostal dual L-TAP blocks in patients undergoing laparoscopic cholecystectomy [7], which are identical to those applied in OPMICS. Figure 1 indicates that the effects of US-TAP and L-TAP in OPMICS may be related to the incisions made for bowel extraction. The subcostal dual L-TAP block, applied by the surgeon, may provide better analgesia for supraumbilical transverse incisions, and the posterior US-TAP block, applied by the anaesthesiologist, for Pfannenstiel incisions. This might explain why OPMICS did not achieve the predetermined minimal clinically important difference.

**Fig. 1:**
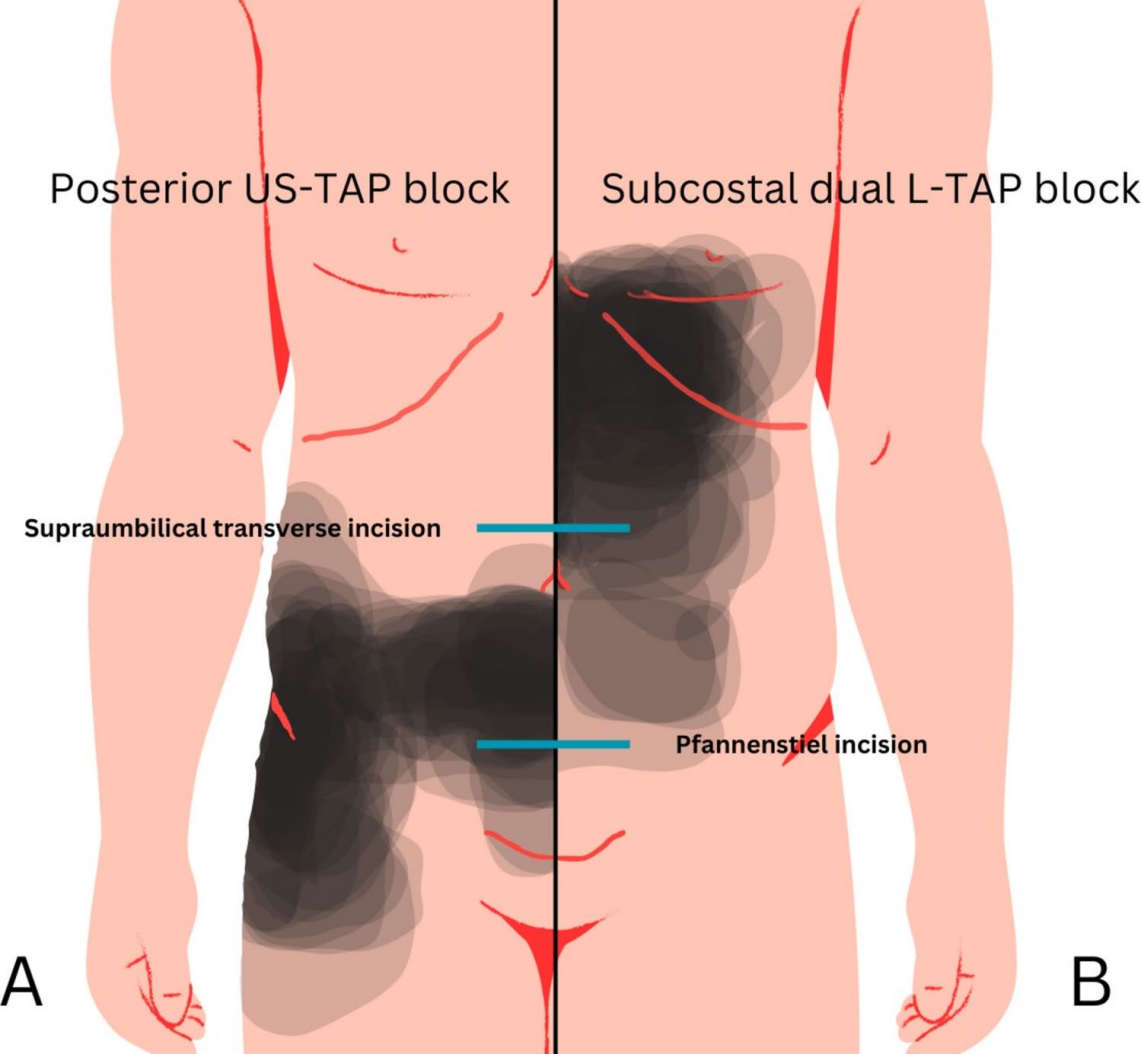
Location of the Pfannenstiel incisions and the common location of the supraumbilical transverse incision with the demarcation of the cutaneous sensory block area of the (**A**) posterior US-TAP block based on data from Støvring et al. [5] and (**B**) the subcostal (dual) L-TAP block from Salmonsen et al. [7]. *L-TAP* laparoscopic-assisted transversus abdominis plane, *US-TAP* ultrasound-guided transversus abdominis plane

This post hoc sensitivity analysis of data from OPMICS investigated whether the two frequently used extraction site incisions (supraumbilical transverse or Pfannenstiel) impacted the clinical analgesic effect of the US-TAP and L-TAP blocks in minimally invasive colon surgery.

## Methods

### Study design

A post hoc sensitivity analysis of using the per-protocol data from the OPMICS trial, a multicentre, patient-, clinician-, and investigator-blinded, placebo-controlled, three-arm randomised clinical trial [9]. The CONSORT statement was used for reporting [10].

We chose to perform a sensitivity analysis for this study to examine the robustness of our findings in OPMICS and to determine how they were affected by the variable ‘type of incision’, i.e. location. The study was conducted as a post hoc analysis for several reasons. Firstly, the CSBA of the subcostal L-TAP had not been assessed, and the differences in CSBA distribution of the blocks had not been considered when planning the OPMICS trial. Finally, including assessment of the CSBA in OPMICS was not an option as it would have unblinded the randomisation.

Ethical approval for the trial was granted by the Regional Committee on Health Research Ethics and the Danish Health and Medicines Authorities under the references H-20026773 and EudraCT 2020-001054-22. The trial was prospectively registered with ClinicalTrials.gov (NCT04311099). Data management was performed in accordance with the General Data Protection Regulation of the European Union and Danish legislation and approved by The Regional Data Protection Agency. All data were stored using a REDCap database [11, 12].

### Study population and randomisation

All 360 patients provided oral and written informed consent. OPMICS was initiated in January 2021, and inclusion concluded in February 2024. Inclusion criteria were patients aged 18 years and older undergoing elective minimally invasive surgery for colon cancer or adenoma with curative intent without a planned ostomy. Exclusion criteria were allergy to LA, liver failure Child–Pugh Score C, body weight less than 40 kg, concurrent pain conditions or weekly intake of analgesics above Step I according to the World Health Organization analgesic ladder, non-compliance due to a language barrier or psychiatric disease, pregnancy, history of inflammatory bowel disease, prior open abdominal surgery featuring a midline or supraumbilical abdominal incision exceeding 8 cm, incisional hernia, or abdominal wall musculature resection.

**Fig. 2:**
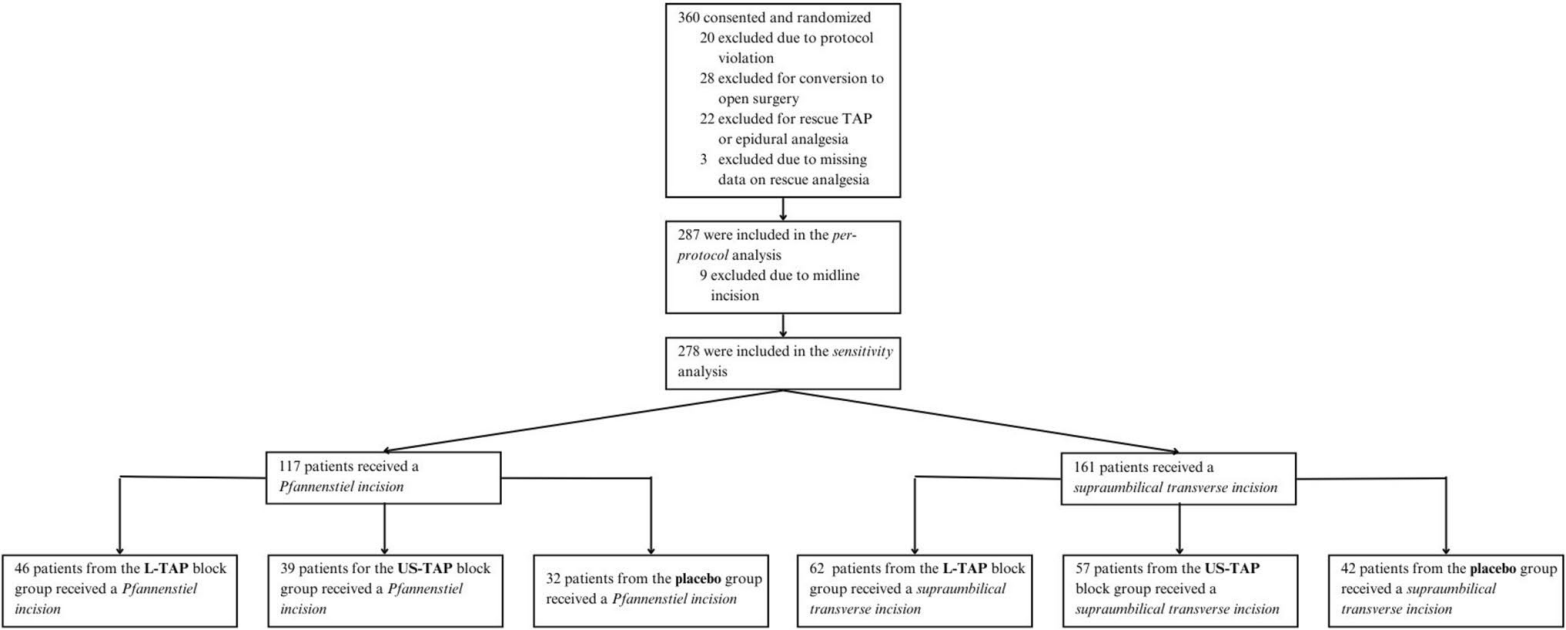
Flow diagram for this sensitivity analysis. *TAP* transversus abdominis plane, *L-TAP* laparoscopic-assisted subcostal TAP, *US-TAP* ultrasound-guided posterior TAP

Patients at each participating hospital were randomly assigned to one of three groups: (1) active posterior US-TAP and subtotal placebo L-TAP; (2) placebo posterior US-TAP and active subcostal dual L-TAP; or (3) posterior placebo US-TAP and subcostal placebo L-TAP in a 3:3:2 ratio without any other stratification. Patients were excluded from analysis if they were converted from minimally invasive to open surgery, if a situation arose in which surgery could not be completed, or if a protocol violation occurred that resulted in no valid data on the primary outcome being reported (Fig. 2).

### Interventions

As a pragmatic study, surgical procedures adhered to the guidelines established by participating hospitals and were carried out at the discretion of the attending surgeon. All sites primarily performed laparoscopic excisions, however, one site also performed robotic-assisted colon surgery in some cases. All sites performed complete mesocolic excision, but in some cases, the central resections may have been more limited. A Pfannenstiel incision is typically used for bowel extraction in left-sided hemicolectomies and sigmoidectomies, whilst a supraumbilical, often muscle-sparing, transverse incision is often used by Danish surgeons in right-sided hemicolectomies with the assumption of less postoperative pain and a lower risk of incisional hernia [13].

Consistent with the study protocol, no local infiltration analgesia was administered at port sites. However, all patients received infiltration analgesia around the extraction site, with a standardised dose of 40 ml of ropivacaine (1 mg/ml) administered at the conclusion of surgery.

All blocks applied contained either ropivacaine (2 mg/ml) or isotonic saline (placebo). For the US-TAP block, 20 ml of ropivacaine (2 mg/ml) or placebo was applied on each side. Two injections of 10 ml (amounting to 20 ml) of ropivacaine (2 mg/ml) or placebo were applied on each side for the L-TAP block. All patients received both a US-TAP block and an L-TAP block. The US-TAP block was performed bilaterally using the posterior approach [5, 14]. The posterior approach targets the TAP approximately 1 cm anterior to the fascia layers tapering into the thoracolumbar fascia. The L-TAP block was performed immediately after placing the first port using a medial/upper and lateral/lower subcostal TAP block approach (dual-TAP block) [7, 15]. The techniques and approaches used to apply the TAP blocks, including relevant differences, are further described in Table 1.

**Table 1:** Transversus abdominis plan block techniques and approaches used in the OPMICS trial

	Posterior ultrasound-guided TAP block	Subcostal dual laparoscopic-assisted TAP block
Technique	Injection under direct visual ultrasound guidance, with distinct visualisation of fascial separation upon injection	Injection under laparoscopic guidance with no direct visualisation of fascial separation (blind). Confirmation of the ‘correct plane’ was visualised by the formation of Doyle’s bulge representing the transversus abdominis muscle covered by the abdominal wall fascia and parietal peritoneum lifted by the local anaesthetic bolus injection
Approach	A linear probe was placed perpendicular to the body’s longitudinal axis in the patient’s flank a few cm cranial to the iliac crest. An 80–120 mm, 21-gauge needle was used, and the injection was carried out in the anterior–posterior direction in parallel with the transducer using hydro-dissection to verify the correct needle position. Targeting the transversus abdominis plane approximately 1 cm anterior to fascia layers tapering into the thoracolumbar fascia	A 21-gauge, 2.5-inch needle was used. A medial subcostal injection was performed between the midclavicular and the central sternal lines and a lateral subcostal injection between the midclavicular and the anterior axillary lines. The needle tip was visualised laparoscopically, passing into preperitoneal fat without perforating the parietal peritoneum. The needle was then withdrawn until the tip was assumed to be located between the posterior rectus sheath and the transversus abdominis fascia (approximately 2–3 mm, more in obese patients), and the injection was performed
Injectate	One injection of 20 ml of 2 mg/ml ropivacaine was administered bilaterally (40 ml, 80 mg in total)	Two injections of 10 ml of 2 mg/ml ropivacaine were administered bilaterally (40 ml, 80 mg in total)
Expected CSBA	Heterogeneous non-dermatomal distribution located to the hip and infraumbilical abdomen	Heterogeneous non-dermatomal distribution located to the supraumbilical abdomen and epigastrium
Expected associated incision	Pfannenstiel incision	Supraumbilical transverse incision

Description of block techniques, approaches, and expected differences

*OPMICS* optimal peripheral nerve block after minimally invasive surgery, *TAP* transversus abdominis plane

Postoperative pain management consisted of oral paracetamol and supplementary IV and oral opioids, which were administered on a patient-controlled basis according to individual needs.

The typical location of the supraumbilical transverse and Pfannenstiel incisions is shown in Fig. 1.

### Outcomes and measures

The primary outcome measure was total morphine dose equivalents (intravenously administered, in mg) in the first 24 h, counting from admission to the postanaesthetic care unit. Using this outcome and based on the patient population, two groups were analysed based on incision location. The groups were analysed with stratification by incision and by TAP block approach for the primary and secondary outcomes. The following secondary outcomes were analysed: NRS pain scores (0–10, no pain to worst possible pain) during rest and activity; incidence of postoperative nausea (0 = no nausea, 1 = mild, 2 = moderate, 3 = severe) and vomiting (0 = no vomiting, 1 = once, 2 = two to three times, 3 = more than three times); mobilisation (1 = without assistance, 2 = needs a little assistance, 3 = needs a lot of help, 4 = cannot be mobilised); quality of recovery (QoR-15 questionnaire, score 0–150), all measured using a postoperative questionnaire completed on postoperative day one between 08:00 and 10:00. The QoR-15 questionnaire was also completed preoperatively. The 30-day postoperative complications according to the Clavien-Dindo classification (as no complications, Clavien–Dindo grade I–IIIa, Clavien–Dindo grade IIIb–IVb, or Clavien–Dindo grade V) and length of stay were also analysed.

### Statistical analysis

As a post hoc sensitivity analysis, no sample size calculation was performed, and findings should be interpreted as exploratory and hypothesis-generating.

All analyses were performed using R statistical software, version 4.2.3 [16]. All available data were used, and no imputations were performed. Continuous data are presented as median and interquartile range (IQR) and categorical data as frequencies and proportions. All primary outcomes were continuous, analysed using linear regression, and presented as absolute differences. Secondary outcomes included categorical variables, which were analysed using Fisher’s exact test. Residual diagnostics assessed statistical model assumptions. For this sensitivity analysis, a *p*-value less than or equal to 0.05 was considered statistically significant when evaluating the outcomes.

## Results

Two hundred eighty-seven patients were included in the per-protocol analysis. After excluding nine patients from the sensitivity analysis due to having received a midline incision after minimally invasive surgery, 161 patients with supraumbilical transverse incisions and 117 patients with Pfannenstiel incisions were included (Fig. 2).

As the type of procedure and whether the anastomosis was performed intra-abdominally or extra-abdominally determined the choice of incision, the distribution of procedures consequently differed between the two incision groups. Similarly, as the groups were not randomised based on incision location, some statistical differences were present between the two groups concerning age, hospital anxiety and depression scale (HADS) score, total intravenous anaesthesia (TIVA), and time from posterior US-TAP block application to start of surgery. Patient characteristics are summarised in Tables 2 and 3.

**Table 2:** Clinical and surgical characteristics of the patients, stratified by incision

	Pfannenstiel incision	Supraumbilical transverse incision	*p*-value
	(*N* = 117)	(*N* = 161)	
Sociodemographic characteristics			
Median age (IQR) in years	70.6 (59.6–78.8)	74.1 (65.0–78.8)	0.04
Sex, *n* (%)			0.40
Male	65 (55.6)	80 (49.7)	
Female	52 (44.4)	81 (50.3)	
Median weight (IQR) in kg	79.0 (70.0–90.0)	75.0 (65.0–90.0)	0.28
Median BMI (IQR) in kg/m^2^	26.0 (23.6–28.7)	25.1 (22.8–29.0)	0.38
ASA classification, *n* (%)			0.15
1	33 (28.2)	35 (21.7)	
2	62 (53.0)	83 (51.6)	
3	21 (17.9)	43 (23.7)	
4	1 (0.9)	0 (0)	
Median HADS score (IQR) [17]	10 (7.3–14.8)	9 (6–12)	< 0.01
Median PCS score (IQR) [18]	9.5 (5–15.8)	9 (5–17)	0.86
Median QoR-15 score—preoperative (IQR) [19]	130 (119–142)	133 (121–142)	0.30
Tobacco, current use, *n* (%)	9 (7.7)	13 (8.1)	1.00
Alcohol, > 7/14 (women/men) units/week, *n* (%)	8 (6.8)	18 (11.2)	0.30
Surgical characteristics			
Type of resection, *n* (%)			< 0.001
Ileocecal resection	0 (0)	3 (1.9)	
Right-sided hemicolectomy	17 (14.5)	102 (63.4)	
Extended right-sided hemicolectomy	3 (2.6)	43 (26.7)	
Transverse colectomy	0 (0)	1 (0.6)	
Subtotal right-sided colectomy	2 (1.7)	5 (3.1)	
Left-sided hemicolectomy	17 (14.5)	3 (1.9)	
Sigmoid resection	75 (64.1)	1 (0.6)	
Other segmental colon resection	0 (0)	2 (1.2)	
Colectomy	0 (0)	1 (0.6)	
Rectum resection	3 (2.6)	0 (0)	
Secondary colon resection, *n* (%)	1 (0.9)	1 (0.6)	1.00
Secondary organ resection, *n* (%)	7 (6.0)	10 (6.2)	1.00
Intraoperative organ lesions, *n* (%)	4 (3.4)	1 (0.6)	0.17
Median surgery time (IQR) in min	170 (140–209)	176 (147–220)	0.40
Stoma created, *n* (%)	1 (0.9)	0 (0)	0.42

Data presented as median (IQR) or *n* (%)

*ASA* American Society of Anaesthesiologists, *BMI* body mass index, *HADS* hospital anxiety and depression scale, *PCS* pain catastrophizing scale, *QoR* quality of recovery

**Table 3:** Anaesthesiological characteristics and postoperative complications of the patients, stratified by incision

	Pfannenstiel incision	Supraumbilical transverse incision	*p*-value
	(*N* = 117)	(*N* = 161)	
Anaesthesiologic characteristics			
Total intravenous anaesthesia, *n* (%)	99 (84.6)	156 (96.9)	< 0.001
TAP block procedure, *n* (%)			0.95
US-TAP block	39 (33.3)	57 (35.4)	
L-TAP block	46 (39.3)	62 (38.5)	
Placebo	32 (27.4)	42 (26.1)	
Median US-TAP block procedure time (IQR) in min	10.0 (8.0–13.3)	10.0 (8.0–12.0)	0.36
Median L-TAP block procedure time (IQR) in min	2.0 (2.0–3.0)	2.0 (2.0–3.0)	0.35
Median time between US-TAP and surgery start (IQR) in min	29.5 (22.0–45.0)	39.0 (30.8–49.3)	< 0.001
Median time between surgery start and L-TAP (IQR) in min	4.0 (3.0–6.0)	4.0 (3.0–6.0)	0.45
Postoperative characteristics			
Postoperative day one questionnaire			
Median NRS pain score when at rest (IQR) from 0 to 10	3.0 (2.0–5.0)	3.0 (2.0–5.0)	0.61
Median NRS pain score when coughing (IQR) from 0 to 10	5.0 (3.0–7.0)	5.0 (4.0–8.0)	0.13
Median QoR–15 score—postoperative (IQR)	102 (86–122)	103 (88–116)	0.55
Postoperative nausea, *n* (%)			0.20
No nausea	78 (67.2)	97 (63.0)	
Mild nausea	24 (20.7)	44 (28.6)	
Moderate nausea	6 (5.2)	9 (5.8)	
Severe nausea	8 (6.9)	4 (2.6)	
Postoperative vomiting, *n* (%)			0.43
No vomiting	86 (74.1)	123 (79.9)	
Once	9 (7.8)	14 (9.1)	
Two–three times	14 (12.1)	11 (7.1)	
Three times or more	7 (6.0)	6 (3.9)	
Mobilisation, *n* (%)			0.87
No assistance	80 (69.0)	105 (68.2)	
Need a little assistance	27 (23.3)	33 (21.4)	
Need a lot of help	8 (6.9)	13 (8.4)	
Cannot be mobilised	1 (0.9)	3 (1.9)	
Median length of stay in hospital (IQR) in days	3.0 (2.0–4.0)	3.0 (2.0–4.0)	0.38
Postoperative complications, *n* (%)			0.39
No complications	108 (93.1)	155 (96.3)	
Clavien–Dindo grade I–IIIa [20]	4 (3.4)	6 (3.7)	
Clavien–Dindo grade IIIb–IVb	3 (2.6)	0 (0)	
Clavien–Dindo grade V	1 (0.9)	0 (0)	

Data presented as median (IQR) or *n* (%)

*TAP* transversus abdominis plane, *US­TAP* ultrasound-guided TAP, *L-TAP* laparoscopic-assisted TAP, *NRS* numeric rating scale, *QoR* quality of recovery

**Fig. 3:**
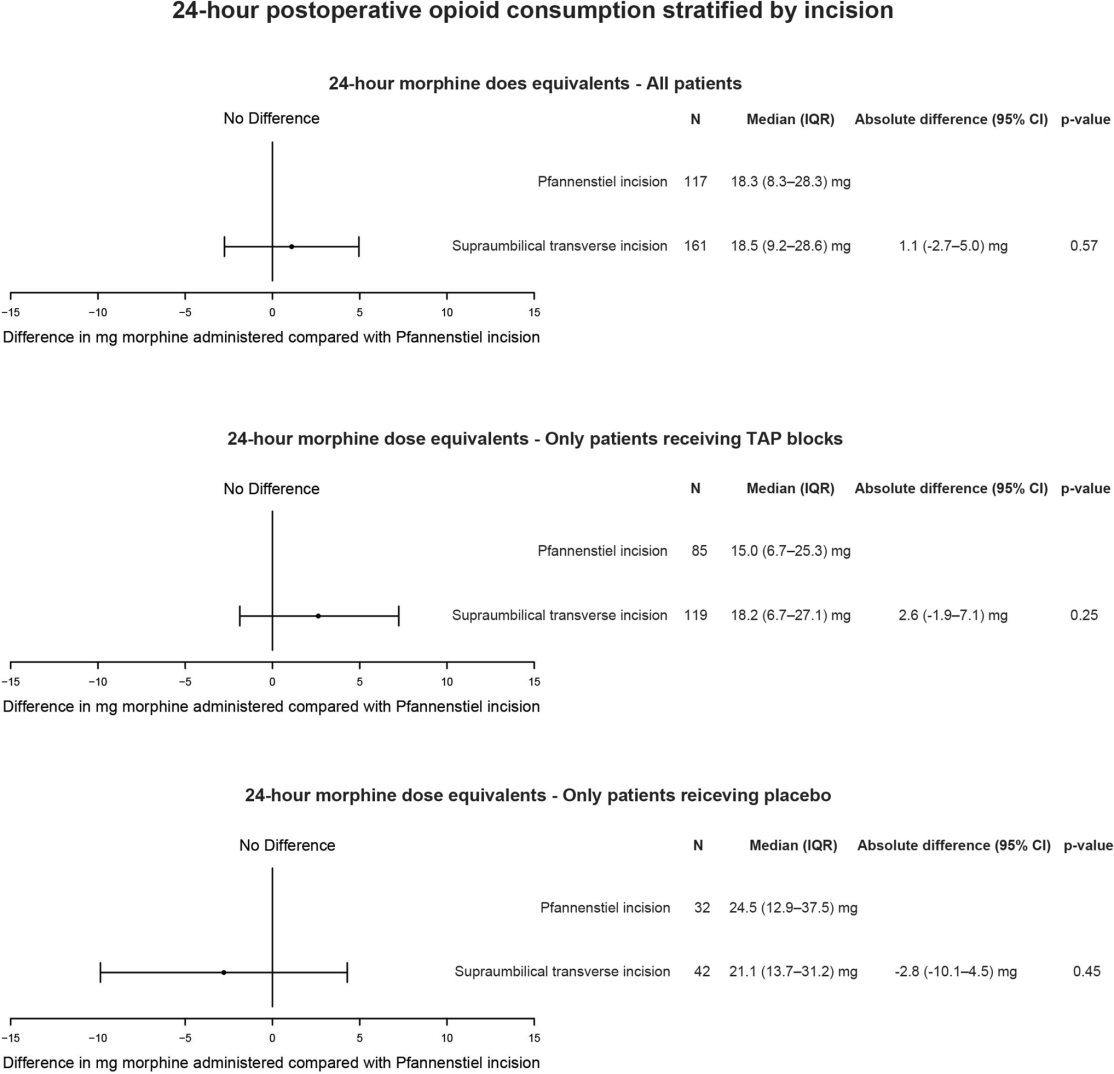
24-h morphine dose equivalents (mg) in patients stratified by incision used for bowel extraction presented for all patients, the laparoscopic-assisted or ultrasound-guided transversus abdominis plane block groups pooled, and the placebo group only. 24-h morphine dose equivalents were analysed by linear regression and presented as the absolute difference with 95% CI. *CI* confidence interval, *IQR* interquartile range

The median 24-h total morphine dose equivalents were 18.5 mg (IQR, 9.2–28.6 mg) after supraumbilical transverse incisions and 18.3 mg (IQR, 8.3–28.3 mg) after Pfannenstiel incisions with an absolute difference of 1.1 mg (95% CI, − 2.7 to 5.0 mg; *p* = 0.57) (Fig. 3).

There was no significant difference between the two incisions for all patients, for patients receiving the TAP blocks, or in the placebo group (Fig. 3). No significant differences were found for secondary outcomes (Tables 2, 3).

### 24-h total morphine dose equivalents stratified by TAP block

#### Supraumbilical transverse incision

**Fig. 4:**
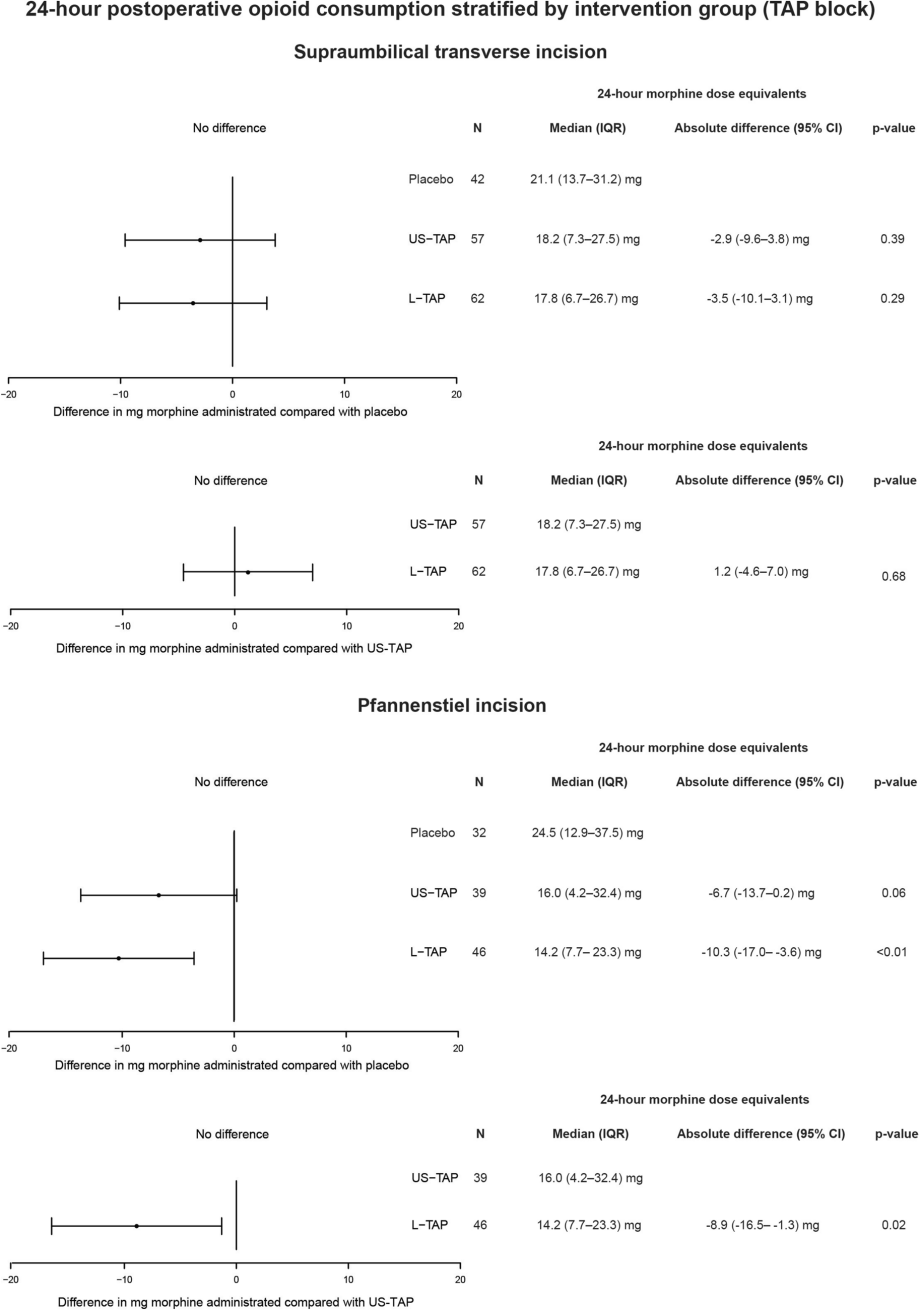
24-h morphine dose equivalents (mg) stratified by US-TAP block, L-TAP block, or placebo for patients with upper transverse incision and Pfannenstiel incision, respectively. 24-h morphine dose equivalents were analysed by linear regression and presented as the absolute difference with 95% CI. *L-TAP* laparoscopic-assisted dual subcostal transversus abdominis plane block, *US-TAP* ultrasound-guided posterior transversus abdominis plane block, *CI* confidence interval, *IQR* interquartile range

The median 24-h total morphine dose equivalents were 18.2 mg (IQR, 7.3–27.5 mg) in the US-TAP block group, 17.8 mg (IQR, 6.7–26.7 mg) in the L-TAP block group, and 21.1 mg (IQR, 13.7–31.2 mg) in the placebo group. There were no statistical differences between the placebo and each of the two TAP blocks after the supraumbilical transverse incision. Similarly, there were no statistical differences between the two TAP blocks (Fig. 4). No significant differences were found for secondary outcomes.

#### Pfannenstiel incision

The median 24-h total morphine dose equivalents were 16.0 mg (IQR, 4.2–32.4 mg) in the US-TAP block group, 14.2 mg (IQR, 7.7–23.3 mg) in the L-TAP block group, and 24.5 mg (IQR, 12.9–37.5 mg) in the placebo group (Fig. 4). The L-TAP block was statistically superior to placebo with an absolute difference of − 10.3 mg morphine dose equivalents (95% CI, − 17.0 to − 3.6 mg; *p* < 0.01), which was considered above the clinically relevant difference in OPMICS. The L-TAP block was also statistically superior to the US-TAP block, with an absolute difference of − 8.9 mg morphine dose equivalents (95% CI, − 16.5 to − 1.3 mg; *p* = 0.02). There was no statistical difference between the US-TAP block versus placebo (Fig. 4).

Regarding secondary outcomes, there was a statistically significant difference in patient-reported outcome measures, as the median QoR-15 score on postoperative day 1 was 114 (IQR, 98–125) in the L-TAP block and 95 (IQR, 77–112) in the placebo group with an absolute difference of 13 (95% CI, 1.7–24.3; *p* = 0.025). No other secondary outcomes showed statistical significance.

## Discussion

This sensitivity analysis showed the L-TAP block to be superior to both placebo and US-TAP block in patients receiving a Pfannenstiel incision. Furthermore, looking at our previous findings of the CSBA of the L-TAP and US-TAP blocks, there was no association between the coverage of the cutaneous segment of the blocks and incision location on 24-h postoperative opioid consumption.

OPMICS used a triple-blind approach (patient, clinician, and researcher) in a population undergoing minimally invasive colon surgery. This design, combined with the chosen TAP block approaches, provided a unique opportunity to examine a possible association between the expected CSBA and the location of the primary abdominal incision regarding postoperative pain management. To our knowledge, this is the first study to investigate the effects of the CSBA in relation to abdominal incision location. The CSBA was not assessed in OPMICS, as this would have unblinded the randomisation to both patients and clinicians.

The main finding of OPMICS [9] was a statistically significant difference in 24-h morphine equivalent consumption of − 5.9 mg for the L-TAP block versus placebo. This did not meet our pre-trial estimate of the minimal clinically important difference of 10 mg morphine dose equivalents, which is the consensus in the literature on postoperative pain management [21]. Interestingly, when isolating the population to those receiving a Pfannenstiel incision, the difference in postoperative morphine consumption increased to − 10.3 mg for L-TAP block versus placebo. This was also reflected in the patient-reported outcome measures, with a higher QoR-15 score on postoperative day one. No such difference was present for the supraumbilical transverse incision. The effect of the US-TAP block on 24-h morphine consumption was not statistically different from placebo for both supraumbilical and Pfannenstiel incisions. This post hoc sensitivity analysis seems underpowered to assess the effect of the US-TAP block in patients receiving a Pfannenstiel incision, as the difference trended towards being statistically significant and of clinical importance. Further studies aiming to assess the analgesic effect of the subcostal L-TAP block in patients receiving Pfannenstiel incisions seem warranted. These should explore the effect in other minimally invasive surgical fields such as gynaecology and urology, where consensus on TAP block effect is similarly divergent [22–26]. In this context, the use of the US-TAP block seems obsolete due to longer procedure time, need for ultrasound guidance and complexity. The US-TAP block should be reserved for procedures such as caesarean sections, where it has previously been shown to have a clinically relevant effect [27, 28]; however, contrary to the expected CSBA, a subcostal US-TAP block might be preferable for these procedures. Studies have also looked into surgeon-administered blocks under direct visual application through the abdominal wall incision [29].

Dermatomal coverage has traditionally been used to assess the effect of abdominal field blocks such as TAP blocks. However, as we have previously pointed out, the term ‘dermatomal coverage’ is insufficient when describing the effects of these blocks [30]. Firstly, it inadequately reflects the true distribution of sensory loss (i.e., the CSBA), as this often exhibits a more heterogeneous, non-dermatomal pattern [5, 6, 31]. Secondly, no evidence suggests that dermatomal coverage or the CSBA translates to effective pain management [32]. These findings contradict the idea of dermatomal coverage as a measure of effect, as, although the expected CSBA of the subcostal dual L-TAP block does not cover the site of the Pfannenstiel incision, there seems to be an analgesic effect. Selecting the type of TAP block based on the CSBA covering the incision does not directly translate to an analgesic effect.

Whilst the value of CSBA and ‘dermatomal coverage’ for assessing new abdominal field blocks is questionable. Posterior injection techniques, such as the ultrasound-guided quadratus lumborum block, whilst technically more challenging, fail to improve postoperative pain [32–34]. A muscle relaxing effect, as suggested by Støving et al. [5], might explain the reduction in opioid consumption from the subcostal dual L-TAP block in patients with a Pfannenstiel incision, rather than sensory loss.

We found no significant differences in surgical and postoperative outcomes or length of stay between supraumbilical transverse and Pfannenstiel incisions despite the association with procedures performed, i.e., right-sided or left-sided resections. Several studies have investigated postoperative pain differences between midline and transverse incisions in abdominal surgery [35–38]. Some suggest the superiority of transverse incisions [35], whilst others find no difference [36–38]. In minimally invasive colorectal surgery, there appears to be no difference in pain scores between midline and transverse incisions [36, 38].

Limited evidence exists comparing postoperative pain outcomes after supraumbilical transverse and Pfannenstiel incisions in minimally invasive colon surgery. A solitary study, examining the comparative effects of intracorporeal versus extracorporeal ileocolic anastomosis in right-sided hemicolectomies, reported a statistically significant reduction in pain scores on the third postoperative day for patients undergoing Pfannenstiel incisions relative to those with supraumbilical transverse incisions [39]. However, due to the technical aspects, there was a significant imbalance in incision type between the intracorporeal and extracorporeal groups, with 39 of 70 patients in the intracorporeal group receiving a Pfannenstiel incision compared to 1 of 70 in the extracorporeal group. The lower pain scores in patients with a Pfannenstiel incision might be due to unknown factors related to intracorporeal anastomosis [39]. Regarding postoperative pain, the anatomical location of the transverse incision appears to be a subordinate factor in the decision-making process between intracorporeal and extracorporeal anastomosis. Given that incisions in minimally invasive colon surgery are typically of limited dimension (approximately 5–7 cm), their contribution to postoperative pain may be overshadowed by other aetiological factors, such as visceral pain and referred shoulder pain resulting from peritoneal insufflation [32, 38]. Future research should prioritise perioperative protocols aimed at mitigating visceral pain.

### Strengths and limitations

This post hoc sensitivity analysis has some limitations. It was not considered in the sample size calculation of OPMICS, and its findings should be interpreted as exploratory and hypothesis-generating. The CSBA in this sensitivity analyses were based on two previous trials conducted by our group [5, 7]. Although this is a potential source of bias, the TAP blocks in the referenced studies [5, 7] were applied identically to those in OPMICS. The patients were randomised according to US-TAP block, L-TAP block, and placebo. Consequently, there are some statistical differences between the groups when stratified by incision, as mentioned previously.

The observed age difference is consistent with previous findings in Denmark, where patients undergoing sigmoid cancer resection [40] tend to be younger than those undergoing right-sided colon cancer resection [41]. The small difference in HADS score, 10 versus 9, is unlikely to be clinically significant but could be a minor confounder. The time between US-TAP block application and the start of surgery differed by about 10 min between incision types. With an estimated block effect of around 8–10 h, this should not influence the outcome [5].

Fewer patients in the Pfannenstiel group received TIVA (85% vs. 97%). TIVA has been shown to improve complications such as postoperative nausea and vomiting, though no such effect was established on postoperative pain or opioid consumption [42]. We found no statistical difference concerning postoperative nausea or vomiting when stratified by incision. Lastly, the posterior US-TAP block did not reach statistical significance. This was likely due to the smaller sample size in this sensitivity analysis stratified by incision, and statistical significance might have been reached with a greater population.

Despite the limitations, this sensitivity analysis provides valuable knowledge based on high-quality data and sheds light on a subject lacking consensus. The assumption that the analgesic effect is related to TAP blocks’ ‘dermatomal coverage’ appears implausible. Interestingly, when isolating the population to patients having received a Pfannenstiel incision, a clinically significant difference of 10 mg in 24-h morphine consumption was achieved for the subcostal L-TAP block, as well as a superior patient-reported quality of recovery.

## Conclusion

The effect of the transversus abdominis plane block seems to be independent of the distribution of the cutaneous sensory block area of the approach. The laparoscopic-assisted subcostal transversus abdominis plane block, applied by the surgeon, is more efficient and easier to apply, and reduces postoperative pain with a high QoR-15 score. Further research is needed to determine its applicability in minimally invasive surgery.

## Data Availability

Data are available upon reasonable request. After deidentification, individual participant data that underlie the results reported in this article, the statistical analysis plan, and analytic codes will be available 9 months following the article’s publication to researchers who provide a methodologically sound proposal. Proposals should be directed to claus.anders.bertelsen@ regionh.dk. Data requestors must sign a data access agreement to gain access.

## Abbreviations

ASA: American Society of Anaesthesiologists
BMI: Body mass index
CI: Confidence interval
HADS: Hospital anxiety and depression scale
IQR: Interquartile range
LA: Local anaesthetic
LOS: Length of stay
L-TAP: Laparoscopic-assisted transversus abdominis plane
MCID: Minimal clinically important difference
NRS: Numeric rating scale
PCS: Pain catastrophizing scale
QoR: Quality of recovery
TAP: Transversus abdominis plane
TIVA: Total intravenous anaesthesia
US-TAP: Ultrasound-guided transversus abdominis plane

## Declarations

### Transparency declaration

First author, Christopher Blom Salmonsen, affirms that this manuscript is an honest, accurate, and transparent account of the study being reported, that no important aspects of the study have been omitted, and that any discrepancies from the study as planned have been explained.
